# The use of shared haplotype length information for pedigree reconstruction in asexually propagated outbreeding crops, demonstrated for apple and sweet cherry

**DOI:** 10.1038/s41438-021-00637-5

**Published:** 2021-09-01

**Authors:** Nicholas P. Howard, Cameron Peace, Kevin A. T. Silverstein, Ana Poets, James J. Luby, Stijn Vanderzande, Charles-Eric Durel, Hélène Muranty, Caroline Denancé, Eric van de Weg

**Affiliations:** 1 Institut für Biologie und Umweltwissenschaften, Carl von Ossietzky University, Oldenburg, Germany; 2 Department of Horticultural Science, University of Minnesota, St. Paul, MN USA; 3 Department of Horticulture and Landscape Architecture, Washington State University, Pullman, Washington, WA USA; 4 Minnesota Supercomputing Institute, University of Minnesota, Minneapolis, MN USA; 5 Université d’Angers, Institut Agro, INRAE, IRHS, SFR 4207 QuaSaV, Beaucouzé France; 6 Plant Breeding, Wageningen University and Research, Wageningen, The Netherlands

**Keywords:** Plant breeding, Agricultural genetics, Genotyping and haplotyping

## Abstract

Pedigree information is of fundamental importance in breeding programs and related genetics efforts. However, many individuals have unknown pedigrees. While methods to identify and confirm direct parent–offspring relationships are routine, those for other types of close relationships have yet to be effectively and widely implemented with plants, due to complications such as asexual propagation and extensive inbreeding. The objective of this study was to develop and demonstrate methods that support complex pedigree reconstruction via the total length of identical by state haplotypes (referred to in this study as “summed potential lengths of shared haplotypes”, SPLoSH). A custom Python script, HapShared, was developed to generate SPLoSH data in apple and sweet cherry. HapShared was used to establish empirical distributions of SPLoSH data for known relationships in these crops. These distributions were then used to estimate previously unknown relationships. Case studies in each crop demonstrated various pedigree reconstruction scenarios using SPLoSH data. For cherry, a full-sib relationship was deduced for ‘Emperor Francis, and ‘Schmidt’, a half-sib relationship for ‘Van’ and ‘Windsor’, and the paternal grandparents of ‘Stella’ were confirmed. For apple, 29 cultivars were found to share an unknown parent, the pedigree of the unknown parent of ‘Cox’s Pomona’ was reconstructed, and ‘Fameuse’ was deduced to be a likely grandparent of ‘McIntosh’. Key genetic resources that enabled this empirical study were large genome-wide SNP array datasets, integrated genetic maps, and previously identified pedigree relationships. Crops with similar resources are also expected to benefit from using HapShared for empowering pedigree reconstruction.

## Introduction

Pedigree information is of fundamental importance in breeding, genetic studies, genebank germplasm management, and for resolving questions regarding cultivar histories. However, many elite individuals and most diversity germplasm of genebank collections have unknown pedigrees. Additionally, recorded pedigree information is sometimes incorrect, as demonstrated in several studies with apple (*Malus domestica*)^1–3^. Although known ancestors of cultivars and breeding selections of many crops are thought to be closely related because of their recorded or speculated shared historical origins (e.g., refs. ^4,5^), these interrelations are often unknown. There are many reports of pedigrees being validated or reconstructed using genotypic data. Microsatellite markers have historically been the most commonly used genetic marker type used in such pedigree reconstruction studies thanks to their multi-allelic nature (e.g., ref. ^1^). Single nucleotide polymorphisms (SNPs) have been increasingly used because of their high throughput, accuracy, and relatively low costs in combination with their abundance, ubiquitous distribution across genomes, and low allele mutation rates. The recent ease of obtaining dense, genome-wide genotypic data provides great opportunity to identify and differentiate among various types of relationships^6^.

A summary of the methods typically used in pedigree reconstruction studies with SNP data was included in Huisman^7^ and Flanagan and Jones^6^. These methods enable identification of parent–offspring relationships, of more distant pedigree relationships such as grandparent–grandchild, of groups of half- or full-sibs through ungenotyped parents, and the exclusion of any close genetic relationships. They have been employed in ecological studies^8^, with humans(e.g., refs. ^7,9^), in animal breeding (e.g., refs. ^10–12^), and in a range of clonally propagated crops including apple (*Malus domestica*)^2,3,13–18^, sweet cherry (*Prunus avium*)^19–21^, apricot (*Prunus armeniaca*)^22^, grape (*Vitis* spp.)^23–26^, eucalyptus (*Eucalyptus* spp.)^27^, French maritime pine (*Pinus pinaster*)^28^, and potato (*Solanum tuberosum*)^29^. In crops, the methods used in such pedigree reconstruction or pedigree validation studies have mostly been limited to the identification or validation of first degree relationships such as parent–offspring or parent–parent–offspring relationships, although methods used for the identification of second degree relationships, grandparent–grandchild relationships^3,13,16^, and for sib-ship reconstruction^27^ have also been occasionally utilized. However, the identification of more complex relationships is still limited.

Most pedigree reconstruction methods that have been typically used in plants lag the newer methods used for humans, but these newer methods have much potential to identify and validate pedigree relationships in crops more efficiently and of greater complexity than previously accomplished. Typically, the methods used in plants either rely on unlinked SNPs, as in Huisman^7^, or they ignore linkage information entirely and instead rely on the sheer abundance of marker numbers (e.g. ref. ^3^). The inclusion of linkage information with genome-wide genetic markers can enable improved depth of pedigree reconstruction results, as demonstrated in many studies with human SNP data (e.g., refs. ^30–34^). The power of these approaches depended on the resolution of the genotypic data (minor allele frequency and number of polymorphic SNPs per centiMorgan) and whether the available data was unphased or phased, among other factors. The methods used by these studies generally involved identification and evaluation of shared haplotypes between pairs or groups of individuals. Several analyses of shared haplotype information have been reported for crops. For example, Toomajian et al.^35^ demonstrated through shared haplotype data that natural selection for early-flowering alleles had occurred in *Arabidopsis thaliana*. These methods were also used to demonstrate selection signatures in barley^36,37^ and wheat^38^ and related methods to demonstrate selection signatures for wheat in the form of conserved haploblocks has also been reported, along with a discussion on the implications of haploblock lengths^39^. However, these methods have not been adapted for pedigree reconstruction in plants. There are important differences between humans and plants that are relevant to such adaptation. For example, plants can give rise to far more offspring, many can tolerate a far higher level of inbreeding, and many are hermaphroditic, allow self-fertilization, and/or are clonally propagated allowing an individual to occur in multiple generations of a pedigree. Extreme endogamy (continuous interbreeding within a small group) and clonal propagation can result in pedigrees that are more complex than, and sometimes not possible, in humans. The development and implementation of methods using shared haplotype length information for pedigree reconstruction in plants could provide similar depth of insights in their pedigrees as has been done in humans.

The ever-growing SNP array datasets for apple^3,21,40^ and cherry^21,41,42^, as well as high-quality integrated linkage maps available^21,43,44^, make these two clonally propagated, outcrossing crops suitable subjects to test shared haplotype length information as a method to reconstruct plant pedigrees. Additionally, there is a wealth of pedigree relationships already confirmed in prior studies for both crops that can be useful for establishing empirical amounts of haplotype sharing at various kinds of relationships. Improved pedigree information would be useful in breeding, genetic studies, germplasm characterization, and for resolving questions regarding cultivar histories. In both apple and cherry, many ancestors with unknown or partial parentage records are suspected of being full-sibs or half-sibs. These suspicions arise from grouping in genotypic clustering analyses, shared phenotypes, similar geographical origins and intertwined histories, or simply because of the unlikelihood of independent parentage among the hundreds to thousands of cultivars in existence.

The objective of this study was to develop and demonstrate a method that supports complex pedigree reconstruction in plants via (a) efficient calculation of total summed potential lengths of shared haplotypes (SPLoSH) for both unphased and phased genotypic data, (b) empirical determination of the association between the degree of haplotype sharing and actual pedigree relationships in apple and cherry, and (c) demonstration of subsequent pedigree reconstruction in a range of close-relatedness scenarios to serve as case studies.

## Results

### Reference SPLoSH frequency distributions and models

**Fig. 1:**
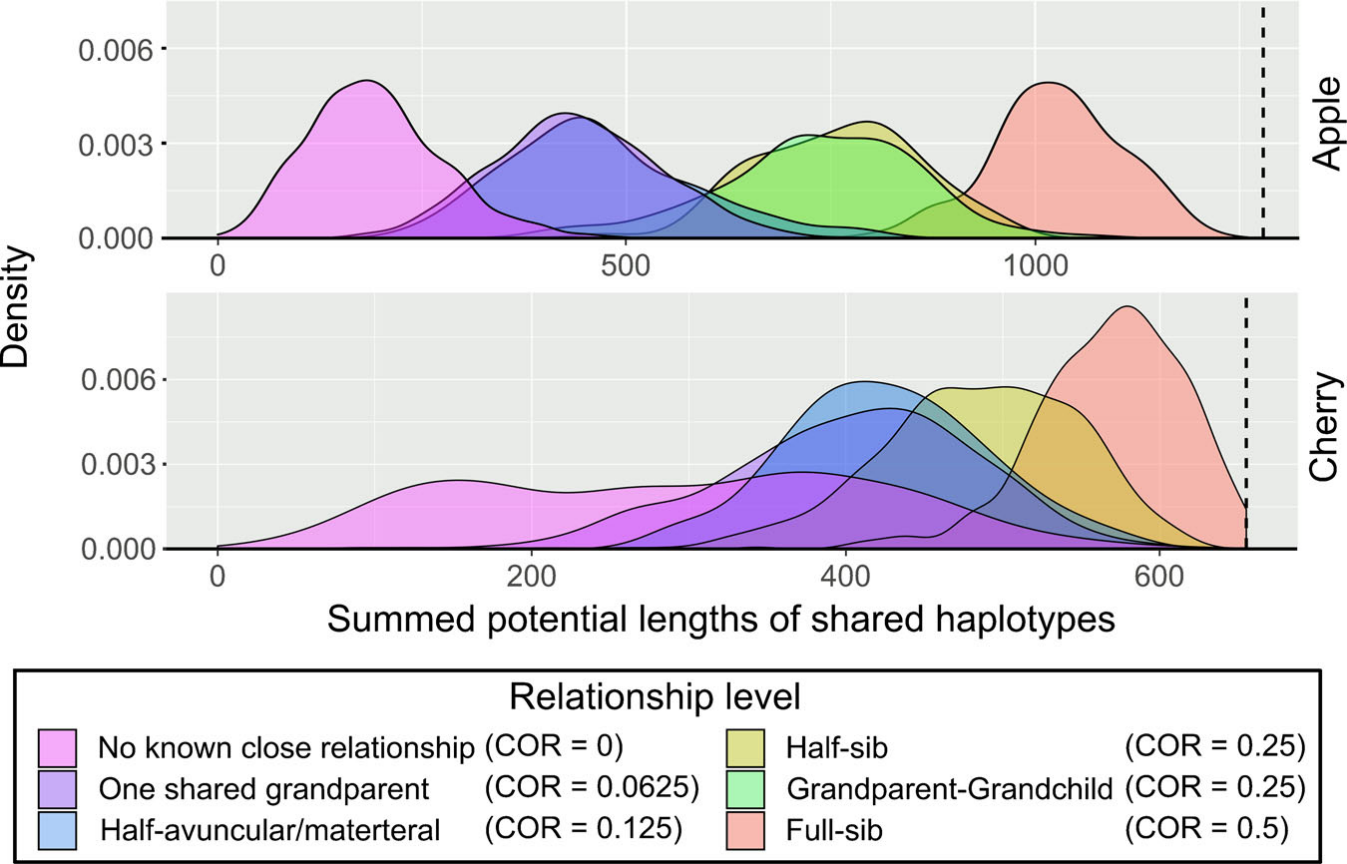
Density plots of summed potential lengths of shared haplotypes for groups of pairs of individuals using phased data. The pedigree relationship levels are distinguished by color for both apple (top) and cherry (bottom). A maximum potential shared haplotype length threshold of 25 cM was used. The vertical dashed lines indicate total lengths of the reference genetic maps of apple and cherry, 1267 and 655 cM, respectively

Distribution means of SPLoSH values for each examined relationship group in apple (accessions listed in Table S1) generally followed the trend of increasing SPLoSH value with increasingly close relationship levels, and distributions were mostly separated according to the different coefficients of relatedness (COR) values of each relationship level (Fig. 1 for the 25 cM threshold, Fig. S1 for all thresholds, and Table S2 for each SPLoSH value per cultivar pair). An exception was observed with the one shared grandparent (OSGP) and half-avuncular/materteral (HAAM) groups (CORs of 0.0625 and 0.125, respectively), which had largely overlapping distributions at the lowest length thresholds (20 and 25 cM), although the means of these distributions were separated at higher thresholds (Fig. S1). The half-sibling (HSIB) and grandparent–grandchild (GPGC) groups (COR of 0.25 for both) had nearly completely overlapping distributions at all length thresholds.

In cherry, distribution means for each relationship group in cherry (accessions listed in Table S3) were less well defined than in apple, but the HSIB and full-sibling (FSIB) groups had clearly distinct distributions compared with other groups (Fig. 1 for the 25 cM threshold, Fig. S2 for all thresholds, and Table S4 for each SPLoSH value per cultivar pair). The observed values associated with peaks of the curves were much higher in cherry than theoretically expected if there were no relationships among recent unknown ancestors (e.g., HSIB: ~500 cM observed in Fig. 1 vs. 328 cM theoretically from the 2 × 656 cM diploid length of the cherry genome). Many NKCR pairs often shared haplotypes in large segments across much of the genome, typically around 1/8 of the genome (e.g., the peak at ~160 cM in Fig. 1) and frequently much higher.

**Fig. 2:**
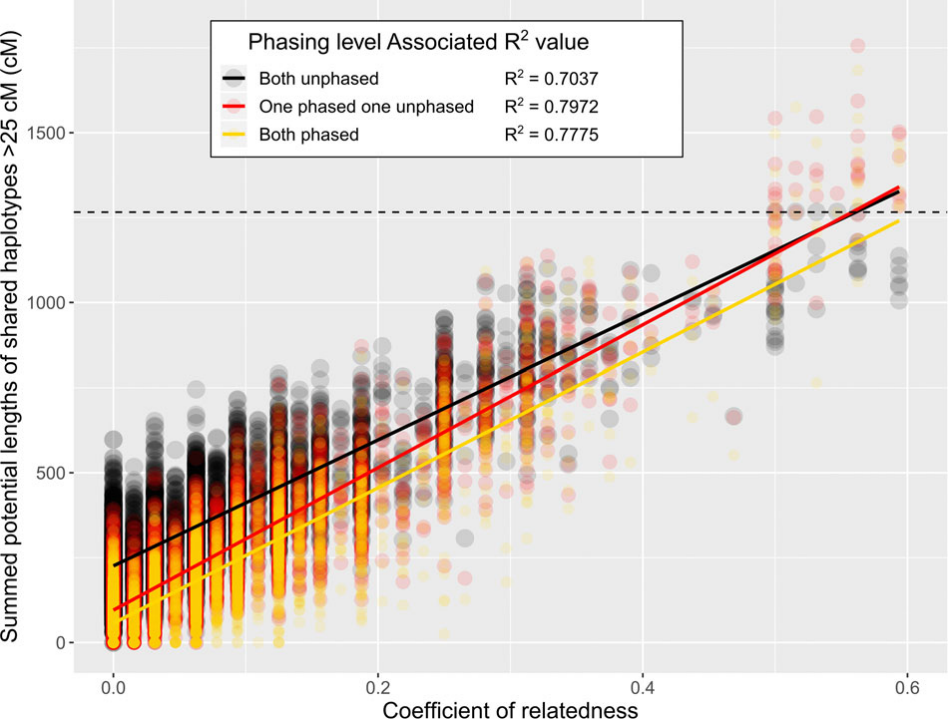
Summed potential lengths of shared haplotypes composed of haplotypes longer than 25 cM vs. coefficients of relatedness. Every pairwise comparison of 116 individuals with known pedigrees up to the grandparent level using either phased data (yellow), unphased data (black), or a combination of both (red) was used to generate the figure. COR values were calculated using pedigree relationships up to the great-grandparent level, where known. The dashed line represents the total genetic length of the apple genome (1267 cM)

SPLoSH values and COR for apple were highly correlated (Fig. 2 and Table S5 for each SPLoSH and COR value per cultivar pair). Having at least one individual phased in comparisons always resulted in models with the highest correlations and higher minimum length thresholds were generally associated with lower observed residual standard errors (Table S6). In cherry, empirically derived quantification of the generalized associations between SPLoSH values and specific relationships resulted in a means of obtaining approximate probabilities of certain relationships (i.e., the Close Relationship Estimator, Table S7) and visualization of the overall pattern (Fig. S3). Just as in the frequency distributions of Fig. 1, FSIB and HSIB relationships were the most distinct (at least ~90% likely to be one of these relationships at ~475 cM and higher, and at least ~90% likely to be FSIB at ~625 cM and higher, 40 cM threshold), while the associations with less-close relationships often blurred with each other.

### Case studies of pedigree reconstruction

#### Case study 1. FSIB relationship (cherry: ‘Emperor Francis’ and ‘Schmidt’)

**Fig. 3:**
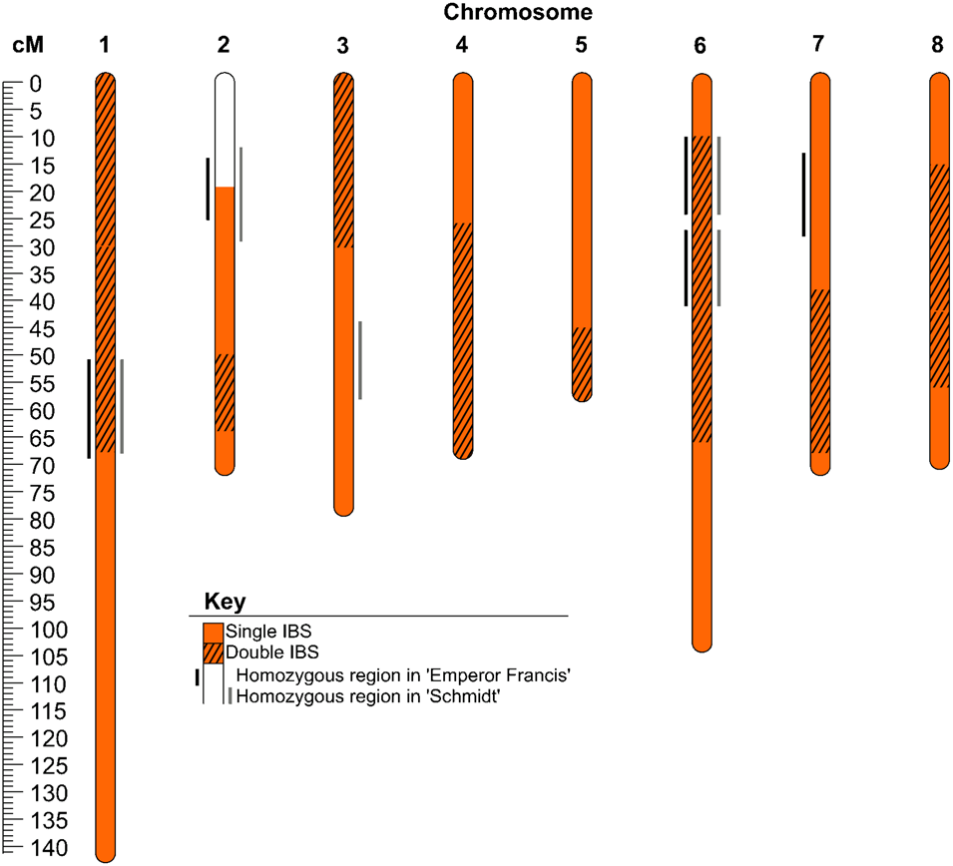
Genomic regions exhibiting single or double IBS matching between ‘Emperor Francis’ and ‘Schmidt’ and/or homozygosity in either cultivar (10 cM threshold used for all)

‘Emperor Francis’ and ‘Schmidt’ had no reported pedigree relationship yet shared 632 cM at all thresholds using unphased data (Table S5), indicative of a FSIB (96% probability) or HSIB (4%) relationship (Table S6). This pair had the highest SPLoSH value in the NKCR group in sweet cherry. Their single identical-by-state (IBS) haplotypes above the 10 cM threshold extended across approximately half (53%) of their genomes, consistent with the expectations of 50% for a FSIB relationship; however, their double IBS accounted for almost all of the other half (45%) of their genomes, much more than the 25% expected for FSIB (Fig. 3). Homozygous regions of at least 10 cM (ranging between 11–18 cM) for each cultivar totaled 11% of their genomes, coinciding in both cultivars for most of that (56 cM, 9% of their genomes) in four regions (Fig. 3).

#### Case study 2. HSIB or GPGC relationship (cherry: ‘Van’ and ‘Windsor’)

**Fig. 4:**
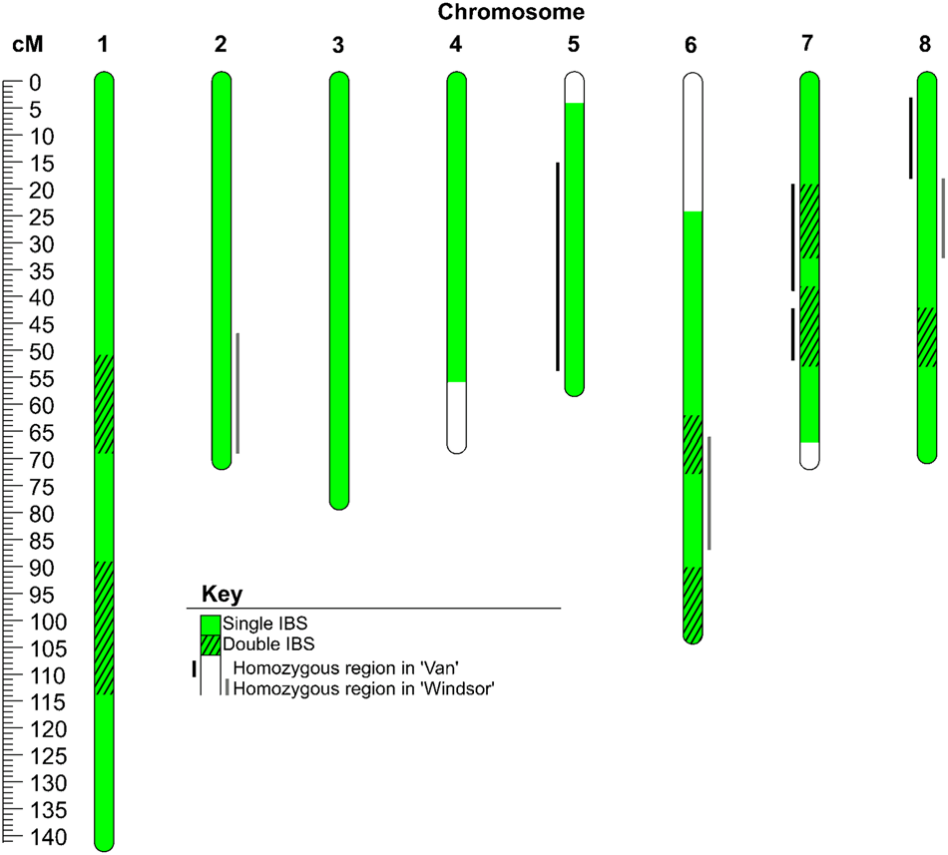
Genomic regions exhibiting single or double IBS matching between ‘Van’ and ‘Windsor’ and/or homozygosity in either cultivar (10 cM threshold used for all)

‘Van’ and ‘Windsor’ had no reported pedigree relationship yet shared 611 cM at all thresholds using unphased data (Table S3). This was the second highest SPLoSH value in the NKCR group in sweet cherry. The very high degree of haplotype sharing was indicative of a FSIB (89% probability) or HSIB (11%) relationship (Table S6). A FSIB relationship was ruled out because the known and available paternal parent of ‘Van’, ‘Black Republican’, did not match as a parent of ‘Windsor’. A HSIB relationship (or GPGC, which has the same COR but this relationship was not modeled for cherry) between ‘Van’ and ‘Windsor’ was therefore considered the likely closest relationship, although single IBS haplotypes shared between these cultivars extended across most (78%) of their genomes, considerably more than the 50% expected for a HSIB relationship (Fig. 4). Furthermore, another 16% of their genomes exhibited double IBS, whereas no double IBS would be expected for half-sibs or grandparents–grandchildren unless their other parents shared recent ancestry. Homozygous regions (10–39 cM) totaled 13% of the genome of ‘Van’ and 8% of ‘Windsor’, although no homozygous regions coincided in these two cultivars nor with case study 1 cultivars (Fig. 4). The higher-than-expected SPLoSH and homozygosity levels indicated the presence of close relationships between recent ancestors. This interpretation was supported by the SPLoSH level for ‘Black Republican’ and ‘Windsor’: 262.5 cM at >40 cM.

#### Case study 3. Two grandparents via single missing parent of grandchild (cherry: ‘Stella’)

**Fig. 5:**
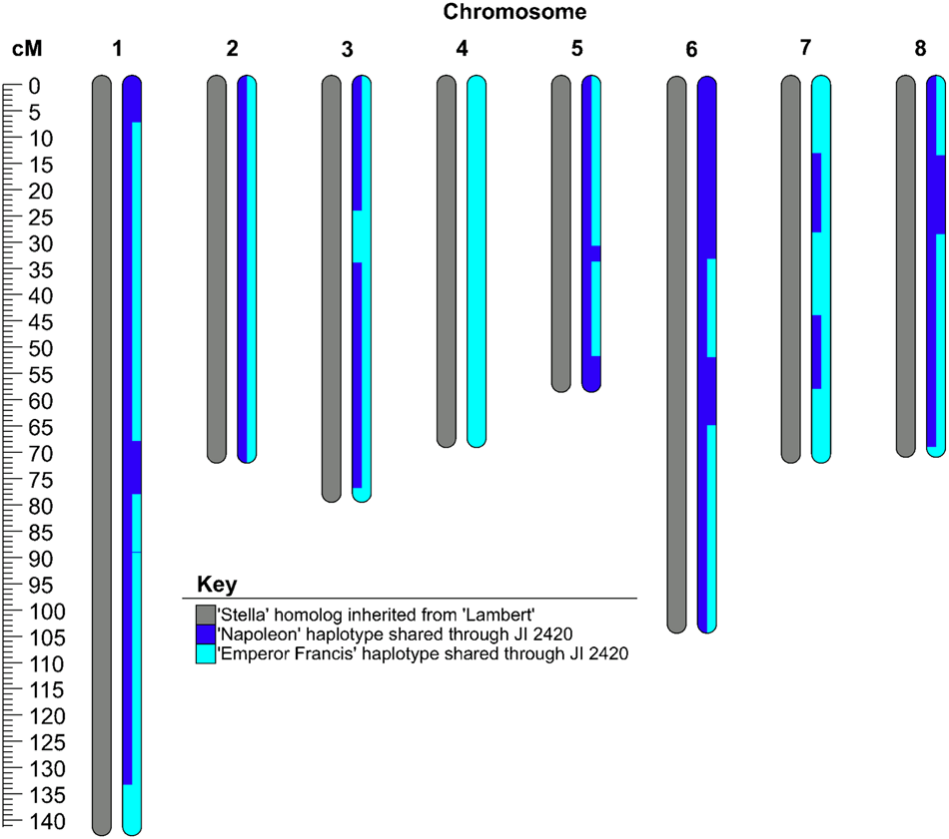
Extended shared haplotypes between deduced paternal homologs of ‘Stella’ (from its ungenotyped parent, JI 2420) and each of the reported paternal grandparents of ‘Stella’, ‘Napoleon’, and ‘Emperor Francis’ (10 cM threshold used for all)

The case of the existing but unavailable parent of ‘Stella’, the self-fertile selection JI 2420, was examined. JI 2420 was reported as being irradiated pollen of ‘Napoleon’ crossed with ‘Emperor Francis’^45^. ‘Lambert’ was previously confirmed as the other parent of ‘Stella’^20^. The homologs of ‘Stella’ deduced to have been inherited from JI 2420 were thus compared to the unphased genotypic data of ‘Napoleon’ and ‘Emperor Francis’. These homologs had the most extended shared haplotypes with ‘Napoleon’ (431 cM), ‘Schmidt’ (356 cM), ‘Emperor Francis’ (342 cM), and ‘Van’ (326 cM). All other cultivars shared less than 274 cM with ‘Stella’, except some offspring of ‘Schmidt’. The pedigree of ‘Stella’ = ‘Lambert’ x (‘Napoleon’ x ‘Emperor Francis’) was devoid of Mendelian inconsistent errors, with all other possible pedigrees having Mendelian inconsistent errors. Also, the unphased genotypic data from ‘Napoleon’ and ‘Emperor Francis’ fully accounted for JI 2420’s contribution to ‘Stella’ with few explanatory recombinations required (Fig. 5), consistent with the recorded pedigree for ‘Stella’.

#### Case study 4. Large HSIB group sharing unknown parent (apple: 29 cultivars via “Unknown Founder 1”)

A group of 29 cultivars was identified that shared between them an average of 783 cM (359–1065 cM) using unphased data (Table S8). Average, minimum, and maximum SPLoSH values had estimated COR values of 0.30, 0.07, and 0.45, respectively. Their genome-wide SNP genotypes allowed the imputation of a hypothetical parent, dubbed “Unknown Founder 1”, for both alleles for 10,172 of 10,252 SNPs (99.2%) (Table S9). All 29 cultivars shared either one of two haplotypes of this Unknown Founder 1 across every chromosome and were all composed of minimally recombined homologs (Table S9). This result was considered confirmation of each individual belonging to the HSIB group.

#### Case study 5. Complex recent ancestry (apple: ‘Cox’s Pomona’)

**Fig. 6:**
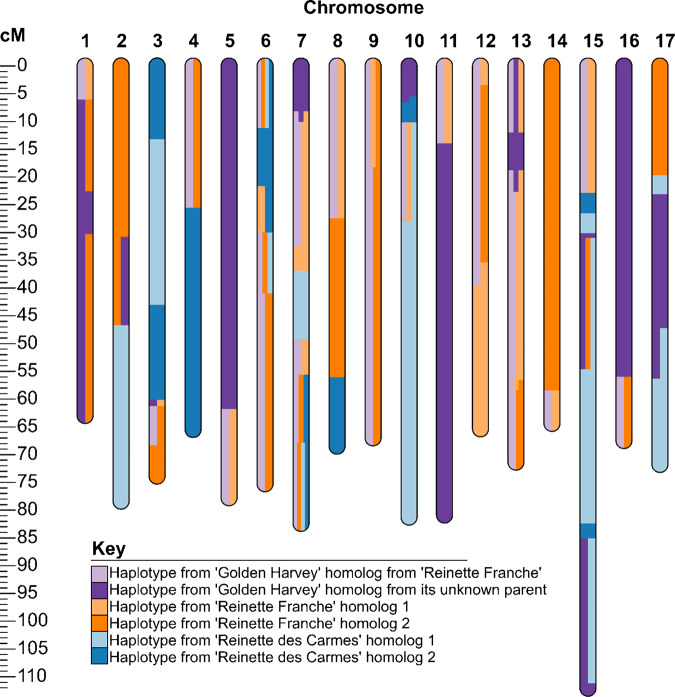
Extended shared haplotypes of the homologs from the unknown parent of ‘Cox’s Pomona’ with its proposed ancestors ‘Golden Harvey’, ‘Reinette Franche’, and ‘Reinette des Carmes’. Areas with more than one color represent regions where multiple haplotypes from these individuals were identical

One parent of ‘Cox’s Pomona’ was previously identified as ‘Alexander’^3^ but the other parent has not been identified. Candidate ancestors were identified for this unknown parent using phased genotypic data ‘Cox’s Pomona’ inherited from this unknown parent. The targeted homologs of ‘Cox’s Pomona’ had SPLoSH values of 747, 566, and 375, respectively, with unphased genotypic data of ‘Golden Harvey’, ‘Reinette Franche’, and ‘Reinette des Carmes’. These cultivars were recorded as being older than ‘Cox’s Pomona’^46^. Extended shared haplotypes from the three candidate ancestors combined completely accounted for the targeted homologs of ‘Cox’s Pomona’ for all 17 chromosomes (Fig. 6). Other cultivars that had SPLoSH values higher than 350 cM only included offspring of these three candidates and no simple combination of them accounted for the targeted homologs of ‘Cox’s Pomona’ for all 17 chromosomes.

The exact contribution of each of the three ancestors of the unknown parent of ‘Cox’s Pomona’ was obscured because of additional genetic relationships among them. ‘Golden Harvey’ was identified as an offspring of ‘Reinette Franche’ and unknown co-ancestry between all three ancestors was evidenced by substantial haplotype sharing between all ancestors (indicated by multiple colors at the same genetic positions on Fig. 6). However, the most likely pedigree was able to be proposed using the following evidence. The relatively smaller total haplotype sharing of ‘Cox’s Pomona’ with ‘Reinette des Carmes’ was consistent with the latter being a great-grandparent of ‘Cox’s Pomona’ rather than a grandparent. Next, ‘Cox’s Pomona’ shared more haplotypes with ‘Golden Harvey’ that were not in ‘Reinette Franche’ (ten, totaling 238 cM) than vice versa (seven, totaling 183 cM) (Fig. 6). Hence the data were consistent with the full pedigree of ‘Cox’s Pomona’ being ‘Alexander’ x [‘Golden Harvey’ x (‘Reinette Franche’ x ‘Reinette des Carmes’)], whereby the direction of each of the crosses was arbitrary.

#### Case study 6. Likely GPGC relationship (apple: ‘Fameuse’ and ‘McIntosh’)

**Fig. 7:**
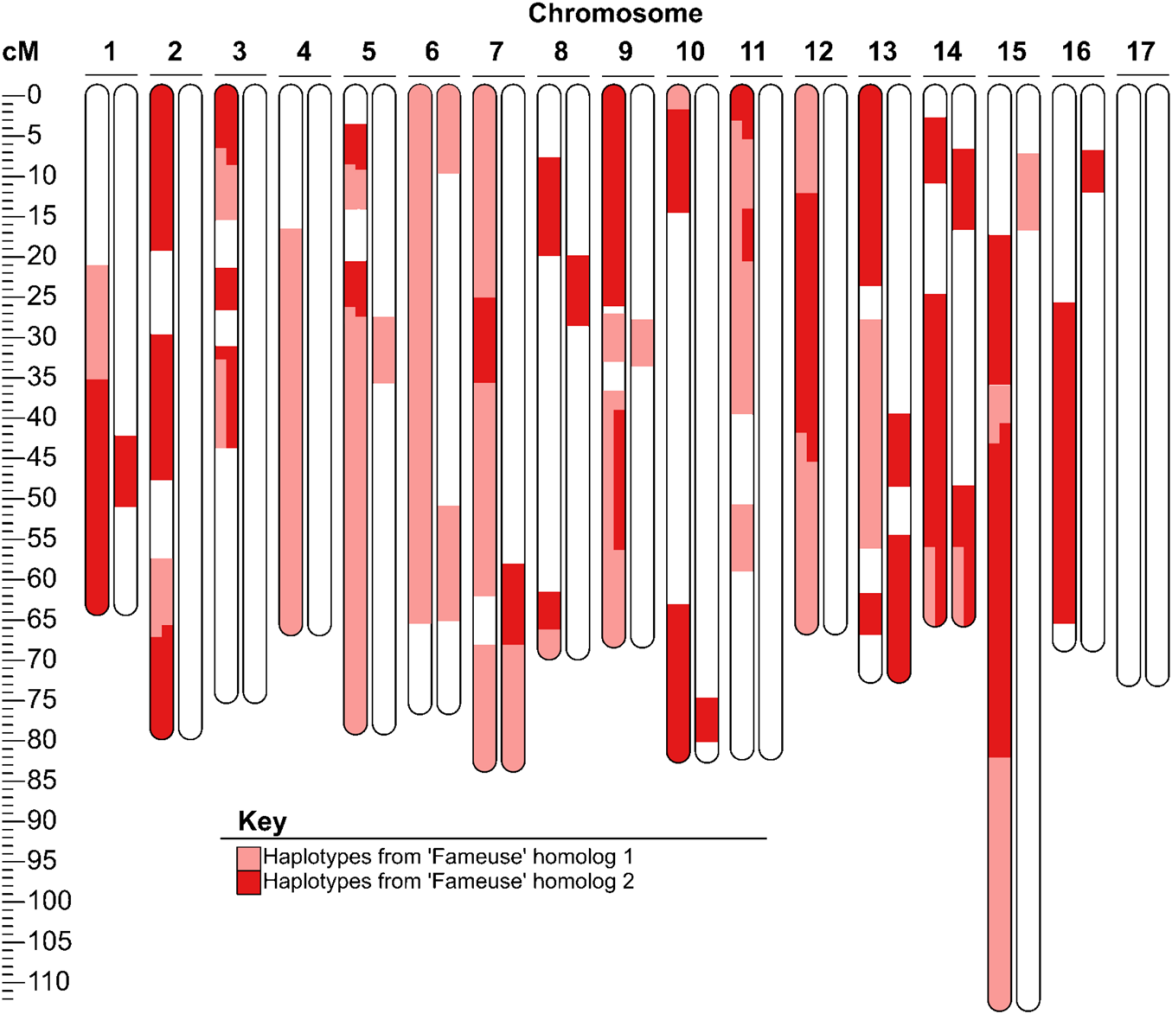
Extended haplotypes greater than or equal to 5 cM from ‘Fameuse’ present in ‘McIntosh’ from phased SNP data. The first homolog for each chromosome pair represents the possible contribution from a hypothetical individual that is both the unknown offspring of ‘Fameuse’ and one parent of ‘McIntosh’. White areas represent haplotypes that the cultivars do not have in common

‘McIntosh’ and ‘Fameuse’ shared 575.8 cM using phased data. This SPLoSH value corresponded with an estimated COR value of 0.26 (Fig. 2 and Table S7). Shared haplotypes often extended over large portions of chromosomes and included 20 chromosome ends, considering only one homolog from each chromosome pair. Portions of the homologs of ‘McIntosh’ showed evidence of being composed of recombinant haplotypes of ‘Fameuse’ on then chromosomes (1, 2, 3, 5, 7, 8, 10, 11, 12, and 15) (Fig. 7) whereas the opposite scenario was only observed twice and with shorter haplotypes on chromosomes 8 and 14. These observations are consistent with ‘McIntosh’ being a grandchild of ‘Fameuse’, though the possibility of an alternative hypothesis, such as ‘Fameuse’ being a double great-grandparent (a scenario not modeled in this study), was not ruled out.

Additional shared ancestry outside of the likely GPGC relationship was evidenced by extended shared haplotypes with ‘Fameuse’ on both ‘McIntosh’ homologs on ten of 17 chromosomes (1, 5, 6, 7, 9, 10, 13, 14, 15, and 16) (Fig. 7), whereby eight chromosomes had shared haplotypes on the same positions of both homologs of ‘McIntosh’. The only candidate ancestor identified for both was ‘Api’, which has been recorded as being older than both^46^. ‘McIntosh’ and ‘Fameuse’ shared 312.5 and 621.9 cM with unphased ‘Api’, which corresponded with estimated COR values of 0.10 and 0.25, respectively. This level of haplotype sharing was consistent with a possible GPGC relationship between ‘Fameuse’ and ‘Api’, but high-quality phased data for ‘Api’ was not available to use for confirming this relationship. While many haplotypes shared between ‘McIntosh’ and ‘Api’ were also shared with ‘Fameuse’, many others were not. For example, ‘McIntosh’ and ‘Api’ shared almost a full homolog of chromosome 17, while ‘McIntosh’ and ‘Fameuse’ had no shared haplotype for this chromosome. Additionally, ‘McIntosh’ and ‘Api’ shared many extended haplotypes, of which shorter fragments were shared between ‘McIntosh’ and ‘Fameuse’, indicating that ‘McIntosh’ inherited these fragments not from ‘Fameuse’, but from another, still unknown descendent of ‘Api’. This was true for chromosome 3, among others, where ‘McIntosh’ shared a haplotype with ‘Api’ from 15.1 cM to the distal end, and which included both short segments ‘McIntosh’ also shared with “Fameuse”.

## Discussion

This study successfully developed and demonstrated a method for efficiently exploiting genome-wide SNP data for identifying then reconstructing the nature of close relationships among cultivars of the outbreeding, perennial, clonally propagated, long-lived crops of apple and sweet cherry. The newly developed Python script HapShared produced a practical measure of genome-sharing among pairs of individuals, SPLoSH, using either unphased or phased genotypic data. Empirically determined associations between this haplotype sharing and known pedigree relationships in apple and cherry successfully established a baseline for estimating close pedigree relationships among any pair of individuals. Subsequent steps of deducing close pedigree relationships using haplotype sharing were demonstrated, which led to numerous discoveries and increased the known relatedness among ancestors of these crops.

Clear and consistent trends of increasing SPLoSH values with increasingly close pedigree relationships were displayed in reference distributions of SPLoSH values generated for known relationships in cherry and apple (Fig. 1 and Figs. S1, S2), the relationship probability estimation model for cherry (Fig. S3 and Table S6), and regressions of SPLoSH values vs. COR values for apple (Fig. 2). These consistent trends demonstrated the applicability of the SPLoSH metric for identifying cases of close relationships. Many of the SPLoSH value distributions for the different relationship groups in apple (Fig. 1 and Fig. S1) and cherry (Fig. 1 and Fig. S2) partially overlapped. Hence, the models built for estimating relationship levels (Fig. 2; Fig. S3; Table S5; and Table S7) were not perfectly discriminating. Nevertheless, patterns were clear enough to successfully use this approach to initially identify close relationships so that the plausible relationships between pairs or among groups of individuals could be deduced in more detailed downstream analyses, as was demonstrated for six case studies.

### Case study results

The case studies presented in this research served to demonstrate the use of SPLoSH information in a range of pedigree reconstruction scenarios. The cultivars chosen for these case studies have had a major impact on breeding and cultivation in cherry and apple, either by themselves or indirectly through their descendants. Discussion of the historic and genetic context of the case studies is provided in File S1. Clarification of the genetic origins of these examined cultivars is expected be of utility for management of genebank collections, breeding decisions, and discovery and validation of marker-trait associations through identity-by-descent (IBD)-based methods.

#### Limitations to pedigree reconstruction methods used: Higher than expected SPLoSH values

A major limitation to our study was the existence of higher-than-expected SPLoSH values for relationships of given COR values. For example, the HSIB group in cherry with a 25 cM threshold averaged 484.9 cM, which far exceeded the expected 327.5 cM for such a relationship. There are several reasons why unexpectedly large SPLoSH values between individuals would be observed:

##### Unknown shared ancestry and endogamy

Pairs of individuals can readily have unknown shared ancestry, as the case studies here indicated. Use of phased data instead of unphased might reduce some issues by avoiding false long shared haplotypes, thereby providing more accurate estimates of the true start and stop positions of shared haplotypes. This is the likely explanation for the observed higher R2 values in the regressions between SPLoSH and COR where at least one individual was phased (Fig. 2 and Table S7). Also, use of phased data enables identification and positioning of recombination events, thereby helping determine the likely seniority of identified ancestors (e.g., case study 5). However, other issues could remain in situations of much distant shared ancestry from multi-generation endogamy. Such inbreeding can result in many short, shared haplotypes, adjacent or overlapping in coupling or repulsion phase, appearing as if they are part of longer extended shared haplotypes. Such patterns were observed in case 6 (Fig. 7), where three extended shared haplotypes separated by unshared haplotypes were observed on chromosomes 3, 9, and 13 (Fig. 7). These observations suggested either multiple triple-, and/or quadruple-recombination events in the gamete from the hypothetical ‘Fameuse’ offspring that formed ‘McIntosh’, or, more likely, that both cultivars have unknown recent shared ancestry.

##### Tendency to conserve a specific haplotype

“Pileup” regions, i.e., long shared haplotypes that many cultivars share, can result from selection or genetic drift. A 29.4 cM region of a fruit size locus on chromosome 2 of cherry^20^ might be associated with such a pileup region due to positive selection for a large-fruit allele. The high frequency of ‘Reinette Franche’ in apple pedigrees^3^ could also be a cause of pileup in apple due to genetic drift.

##### Extended stretches of homozygosity

A third reason could be stretches of homozygosity that exceeded the length thresholds. When calculating SPLoSH values through pairwise comparisons using phased data, such a region was counted double the number of times. Repeated appearance of certain cultivars in the pedigrees of cultivars, such as ‘Reinette Franche’ in apple, would exacerbate the occurrence of homozygosity.

##### Technical limitations of the SNP array used

Some regions of the genome might be insufficiently informative due to low SNP density and/or a lack of informativeness of the SNPs there. Both could lead to the detection of long shared haplotypes that are IBS but not truly IBD. The issue of SNP density was likely a more frequent issue in the cherry dataset (2.4 SNPs/cM) than apple (8.3 SNPs/cM). However, even in the apple dataset there were 30 gaps greater than 2 cM, with the largest being 4.2 cM^44^, indicating that these issues would be present in apple to some degree as well.

### Limitations to pedigree reconstruction methods used: Crop-specific limitations

Some limitations in pedigree reconstruction might be crop-specific. In our study, SPLoSH value distributions for cherry were wider and less well defined than those of apple, which could be because of the lower SNP density, less pedigree information, the less diversity among the tested cultivars, and the lower number of chromosomes in cherry compared to apple. In cherry, the distribution of the NKCR group was particularly wide (Fig. 1 and Fig. S2), as insufficient pedigree information prevented limiting the included individuals to only those with known grandparents as was strictly done for apple.

Requirements for effective pedigree reconstruction are not readily available for many crops. These requirements are a high-quality genetic map, many pedigree-connected individuals, and highly curated SNP datasets for phasing and for building of reference relationship distributions. Additionally, crops with a recent history of hybridization between sexually compatible but otherwise isolated and differentiated species may greatly complicate this type of pedigree reconstruction. However, the use of shared haplotype length data might still be the most efficient way to reveal genetic relationships even if reference relationships are unavailable. For example, even in the absence of known pedigree information, SPLoSH values are expected to be a better proxy for relatedness than simply using SNP data without linkage (and thus recombination) information.

### Comparison to shared haplotype-based pedigree reconstruction in humans

Higher than expected SPLoSH values between pairs of individuals have been noted in human studies on pedigree reconstruction and/or relationship estimation, and have been discussed (e.g., refs. ^32,47^). However, these issues have not prevented the inferring of distant pedigree relationships, as human studies using autosomal haplotypes can identify up to and including 13th-degree relatives^33^. In comparison to humans, there are multiple reasons why these issues might still prevent such high level of relationship prediction in crop data. First, because humans are a far more studied organism and relationship estimation research in humans has already found wider utilization in areas ranging from criminal justice to commercial genotyping services for use in genealogy^47^, the technology has advanced further in areas such as genome quality, SNP choice regarding representation of genetic diversity and robust performance, and affordability of large SNP arrays in humans compared to that of most crops, including apple and cherry. Second, both apple and cherry are hermaphroditic and so can be both mothers and fathers in crosses, are vegetatively propagated, are long living, and can tolerate higher levels of inbreeding than humans. Due to all this, a single individual may occur as parent in multiple generations in each parental lineage of an individual. This likely results in far greater haplotype sharing from both recent and distant relationships in these crops compared to in humans. However, particularly high levels of haplotype sharing in humans arising from multi-generation endogamy have been noted in some human studies (e.g., ref. ^48^), though the extent of this multi-generational endogamy is still far greater in crops. Third, all the individuals evaluated in the present study are cultivars or breeding selections intensely selected by humans. Hence, the opportunities for pileup regions in plants are expected to be much higher than in humans.

### Future needs

Methods are needed for scaling up pedigree reconstruction that avoid laborious manual examination of recombination evidence, for accounting for endogamy, and for confidently confirming HSIB groups without manual imputation of unknown founders. Some of these needs have been met for human data, but the aforementioned limitations and peculiarities of plants need further consideration to expand their application to plants.

## Materials and methods

### Plant material and SNP data

A set of 510 unique diploid apple accessions was used in this study (Table S1). Apple SNP array data for these individuals was obtained from Howard et al.^44^, for which germplasm had been genotyped with either the Illumina apple Infinium™ 20 K SNP array^49^ or the Affymetrix apple Axiom^®^ 480 K SNP array^43^. The SNP data processing and genetic map used were both described in Howard et al.^44^. All 10,295 SNPs deemed to be of high quality by Howard et al.^44^ were included, although only 8412 of these were available from Axiom 480 K SNP array data such that 107 individuals genotyped on the Axiom array and included in this study (indicated in Table S1) had missing data for the remaining 1883 SNPs. Some of the individuals included for the case studies were drawn from the ongoing apple pedigree reconstruction project^40^. All parent-offspring relationships included (Table S1) that were previously known through pedigree records, literature, unpublished results from the FruitBreedomics project^50^, or from the ongoing pedigree reconstruction project described in Howard et al.^40^ were validated via methods described in Vanderzande et al.^21^.

A set of 164 unique diploid sweet cherry accessions was used in this study (Table S2), for which curated, high quality SNP data were available from Vanderzande et al.^21^ for a set of 1617 SNPs from the Infinium cherry 6 K SNP array^51^. Known pedigree relationships and the genetic map used in the present study were those reported in Vanderzande et al.^21^. An additional relationship included for sweet cherry was that ‘Early Burlat’ and ‘Moreau’ being full-sibs, deduced from their high degree of SSR allele sharing^52^ and close affinity revealed by SNP genotypic data in the present dataset. This additional relationship was included a priori because it connected many additional pairs of descendant individuals, thanks to which enough “known relationships” were obtained to generate meaningful SPLoSH reference distributions for use in analyses below.

For both crops, phased SNP genotypic data were generated for particular case studies using FlexQTL™^53^ based on pedigree information listed in Table S1 for apple and in Table S2 for sweet cherry.

### Generation of shared haplotype length information

A custom Python script was created and used to generate lists of shared haplotype lengths between every pair of accessions within each crop given the genetic map used. The script, named HapShared, can handle both phased and unphased genotypic data and also so-called null-alleles. A shared haplotype in HapShared was defined as a region that starts and ends with SNPs where both individuals had at least one allele in common and where each SNP in between also shared at least one allele or had missing data for one or both individuals. The provision for missing data was included to avoid truncation of true shared haplotypes by any occasional missing data. HapShared identifies the start and stop positions of shared haplotype, calculates the length of each shared haplotype, and sums those that pass a user-defined length threshold into the parameter “summed potential lengths of shared haplotypes” (SPLoSH). The term “potential” is included because end points for shared haplotypes might be necessarily estimates due to missing data, gaps in the array, and, in the case of unphased data, the inability to differentiate stretches of haplotypes that are IBS from IBD. The script uses an “*A B C* –” format for SNP genotyping, where the alleles *A* and *B* arise, respectively, from A and T vs. C and G nucleotides from SNP arrays, *C* codes for null alleles, and “-” for missing data. Phased data was imported as doubled haplotypes, i.e., SNPs from phased haplotypes with the *A* allele were changed to *AA* and so on for other alleles.

### Use of HapShared to generate reference SPLoSH distributions for discrete pedigree relationships

#### Apple

Subsets of the SPLoSH information consisting only of pairs of accessions with known or identified relationships (Table S3) were used to determine numbers and lengths of shared haplotypes for various discrete pedigree relationships. The relationship groups were full-sibling (FSIB), HSIB, GPGC, HAAM, OSGP (i.e., half first cousins), and no known close relationship (NKCR). The apple FSIB group comprised 109 pairs of full-sibs from a genetically diverse subset of 137 accessions. All four grandparents were known for each individual in this apple group and there were no common ancestors between the parents of the accessions from each pair up to the great-grandparent level. The apple HSIB group comprised 80 pairs of half-sibs from a diverse subset of 53 accessions, where the shared and unshared parents in each HSIB pair had no known shared ancestors to at least the grandparent level and cases to at least the great-grandparent level. The apple GPGC group comprised 127 pairings between 68 grandchildren and 39 grandparents, with no known further relationships within each pair up to at least the grandparent level for the grandchildren and up to the great-grandparent level for the grandparents. The apple HAAM group comprised 96 pairs of individuals with HAAM relationships from a group of 56 accessions. The pedigrees for each individual in this set were established up to at least the grandparent level, and there were no other known relationships within the pairs up to the great-grandparent level. The apple OSGP group comprised 633 pairs of individuals from a group of 97 accessions. Each pair shared only a single grandparent, with pedigrees identified up to at least the grandparent level and no other known relationship up to the great-grandparent level. The apple NKCR group comprised 1199 pairs of individuals from a group of 123 accessions. The pedigree of each accession in this group was known up to at least the grandparent level and each pair had no known relationship up to the 4x great-grandparent level. Frequency distributions of SPLoSH information were built for each relationship group. Separate distributions were built for each relationship group using shared haplotypes greater than thresholds of 20, 25, 30, 35, and 40 cM.

A separate dataset was constructed for a comparison of SPLoSH information with COR values. These were calculated for all 6670 pairs among a set of 116 accessions with pedigrees known to at least the grandparent level for all four grandparents (Table S4). COR values were calculated using known pedigree relationships up to the great-grandparent level, where known. SPLoSH information (thresholds of 20, 25, 30, 35, and 40 cM) was generated separately for three types of genotypic data available for each pair of individuals: (i) both individuals being unphased, (ii) one individual unphased and the other phased (averaging the values obtained for the two ways this could be arranged), and (iii) both individuals phased. This SPLoSH information was regressed against COR values using R version 3.6.0 (R core Team 2019).

#### Cherry

SPLoSH information was generated for cherry, following the same approach as for apple, for the discrete pedigree relationships of FSIB, HSIB, HAAM, OSGP, and NKCR (Table S5) and using the same criteria of extent of pedigree knowledge except for NKCR. The number of pairs of individuals and number of accessions included for each of the relationships, respectively, were the following: FSIB 585/84; HSIB 750/79; HAAM 74/27; OSGP 518/60; and NKCR 641/51 (Table S5). For NKCR, individuals were included even if parents or grandparents were unknown (otherwise, there would have been no representatives of this relationship for cherry) and only cultivars were considered (i.e., no unselected offspring, unlike for the other relationships). No GPGC relationships were available for cherry because pedigree information was too scarce on grandparents, and there were otherwise ancestors shared between grandparents and grandchildren in other parts of their pedigrees. Frequency distributions of SPLoSH information were constructed for cherry using thresholds of 20, 25, 30, 35, and 40 cM, as for apple. No regression between SPLoSH values and COR values was made for cherry because there was substantially less pedigree information available to make such a comparison. Instead, the cherry SPLoSH information was used to empirically calculate the relative frequency that any given SPLoSH value represented a COR of 0.5, 0.25, 0.125, or 0.0625 (using FSIB, HSIB, HAAM, and OSGP data, respectively). To ensure equal loading for each relationship, calculations were made on random subsets of data consisting of *n* = 74 observations for each relationship, where 74 was the lowest number of observations obtained for any relationship (HAAM), and ten iterations of random sampling were combined for each relationship. Although all shared haplotype length thresholds were considered, only that of 40 cM, the most conservative that gave the closest association with the known relationships, was reported. A sliding window of ±20 cM around each SPLoSH value was used to smooth local variation. Resulting relative frequencies of relationship vs. SPLoSH values were plotted in a line graph using R version 3.6.0 (R core Team 2019). These frequencies were used to estimate the probability of any given SPLoSH value belonging to each of the four relationship types, manifested in a simple Excel-based tool, the Close Relationship Estimator (Table S6).

### Pedigree reconstruction methods and case studies

The obtained insight in SPLoSH information for pairs of individuals with known relationships was used to deduce previously unknown close relationships in three case studies with apple and three with cherry, to demonstrate in a stepwise manner the effectiveness of this information for pedigree reconstruction in plants. The specific case studies covered a range of pedigree reconstruction scenarios and involved increasing levels of phased genotypic data. All pedigree relationships to be reconstructed were beyond parent–offspring among genotyped individuals, as those were and could be readily identified with simpler methods. The first step of this process was to target pairs with the highest SPLoSH values. These SPLoSH values were compared to the empirically derived reference distributions for each crop to establish a hypothesis of the likely relationship level. For apple, this meant comparing the SPLoSH value to the regression between SPLoSH values and COR. A minimum length threshold of 25 cM was used for the apple pedigree reconstruction case studies because it had the most balanced R^2^ and residual standard errors across the phasing and length threshold levels evaluated. For cherry, the Close Relationship Estimator was used (Table S6). The next steps of the process for deducing the relationships depended on the specific scenarios encountered and the level of phased genotypic data available. These steps were applied to larger apple and cherry datasets as part of ongoing pedigree reconstruction studies, with only results relevant to the included case studies described here.

For cases where a full-sib relationship was likely or possible, the genome-wide degree and patterns of double vs. single vs. no IBS were examined using unphased genotypic data, with the lack of phasing representing typical analytical situations of phasing not being possible or not yet done. “Double IBS” refers to the haplotype sharing between both homologs of both individuals, as illustrated in Fig. S4. Theoretically, FSIB relationships are distinguishable from HSIB relationships by observing a combination of a particularly high SPLoSH value and regions with double IBS covering ~25% of genome, assuming no endogamy among parents. Even in the presence of a high degree of double IBS, HSIB relationships could be distinguished from FSIB if an available parent matches one of the individuals but not the other. Pedigree reconstruction for a FSIB relationship (case study 1) and a HSIB relationship (case study 2) were demonstrated.

Additional information was required to distinguish HSIB from GPGC relationships (both being associated with a COR of 0.25 in the absence of endogamy). Some GPGC relationships were confirmed via identification of a second candidate grandparent that together could account for an unknown parent (case study 3), following the method described in van de Weg et al.^16^. This method checks whether a combination of two grandparents could account for the haplotypes from a missing parent of an individual by checking for the presence or absence of Mendelian inconsistent errors. A true instance of two individuals being the grandparents of another individual through an unknown parent would be where no Mendelian inconsistent errors were observed, or where those observed were few and due to either uncalled null alleles or incorrect SNP calls.

Some HSIB relationships were confirmed by identifying a group of individuals with SPLoSH values between all pairs of the group being consistent with a likely HSIB or GPGC relationship, with confirmation of the HSIB relationship being conducted via imputation of the genome-wide genotypes of the hypothetical common parent (case study 4). For this imputation, when the SNP data for the other parent of an individual was available, data for SNPs heterozygous in the prospective half-sib were also used if the known parent was homozygous for that SNP (e.g., half-sib individual = *AB* and known parent = *AA*, deduced allele of the other parent inherited by half-sib = *B*). When the other parent was unavailable in the dataset, only data for SNPs that were homozygous could be used. During imputation, the genome-wide SNP profile for the hypothetical common parent was compared to each individual in the entire putative HSIB group to determine whether one homolog of each chromosome in each putative half-sib was composed of haplotypes from the imputed SNP profile of the hypothetical common parent, allowing for occasional recombination consistent with Mendelian inheritance. This determination was considered confirmation of each individual belonging to the HSIB group.

Alternative and more complex pedigree strategies were necessary for individuals with one or two unknown parents when the unknown parents were unable to be imputed due to a lack of siblings and when both grandparents of an unknown parent were not available in the dataset. In these cases, candidate ancestors, sometimes more distant than GPGC, were identified using SPLoSH information. Candidate ancestors were those that shared relatively high SPLoSH values and were plausible in view of available provenance information. Use of phased genotypic data for an individual was necessary in these cases because phased data was more discerning for identifying close ancestors and could be used to identify recombination evidence to confirm the relationships present (as demonstrated in case studies 5 and 6). More complete pedigree reconstruction for an individual involved identification of candidate ancestors whose phased SNP data accounted for all haplotypes of the individual and where haplotype sharing levels and recombination evidence provided a generation order (case study 5).

Where complete pedigree reconstruction was not possible, partial pedigree reconstruction was attempted. Likely GPGC relationships were made if the following criteria were met: (a) haplotype sharing was consistent with such a relationship; (b) multiple extended haplotypes of the candidate grandparent with evidence of single recombinations were present in the candidate grandchild; and (c) extended haplotypes from the candidate grandparent covered roughly a quarter of the ends of chromosomes of the candidate grandchild (case study 6).

Haplotype sharing across genomes for the case studies were displayed for output from HapShared using a 10 cM threshold for cherry and a 5 cM threshold for apple, rather than the larger thresholds for pedigree reconstruction, to best demonstrate the reality of haplotype sharing in the presence of recombination and thereby to increase power in determining generation order (case 5) and generation distance (case 6). Four shorter shared haplotypes were included in the visualization of case 5 because results suggested that historic recombination had fragmented some haplotypes inherited from grandparents and great-grandparents into haplotypes below the 5 cM threshold.

## Supplementary information

Figure S1

Figure S2

Figure S3

File S1

Figure S4

Table S1

Table S2

Table S3

Table S4

Table S5

Table S6

Table S7

Table S8

Table S9

## Data Availability

The curated apple data is available at the Genome Database for *Rosaceae* - https://www.rosaceae.org/publication_datasets. The curated cherry dataset was previously made available^21^. HapShared can be downloaded with a free license for academic or nonprofit research use by accessing https://z.umn.edu/HapShared.
