# Supplementary material for: The use of shared haplotype length information for pedigree reconstruction in asexually propagated outbreeding crops, demonstrated for apple and sweet cherry
Source: Hortic Res. 2021 Sep 1;8:202. doi: 10.1038/s41438-021-00637-5 (PMC8408172; doi:10.1038/s41438-021-00637-5)

**Supplementary Figure S1**. Density plots of summed maximum potential shared haplotype length data for groups of pairs of individuals with the indicated pedigree relationships using unphased data for apple. Numbers to the right of each graph are thresholds for the minimum shared haplotype lengths used. The vertical dashed line indicates the total length of the reference genetic map.


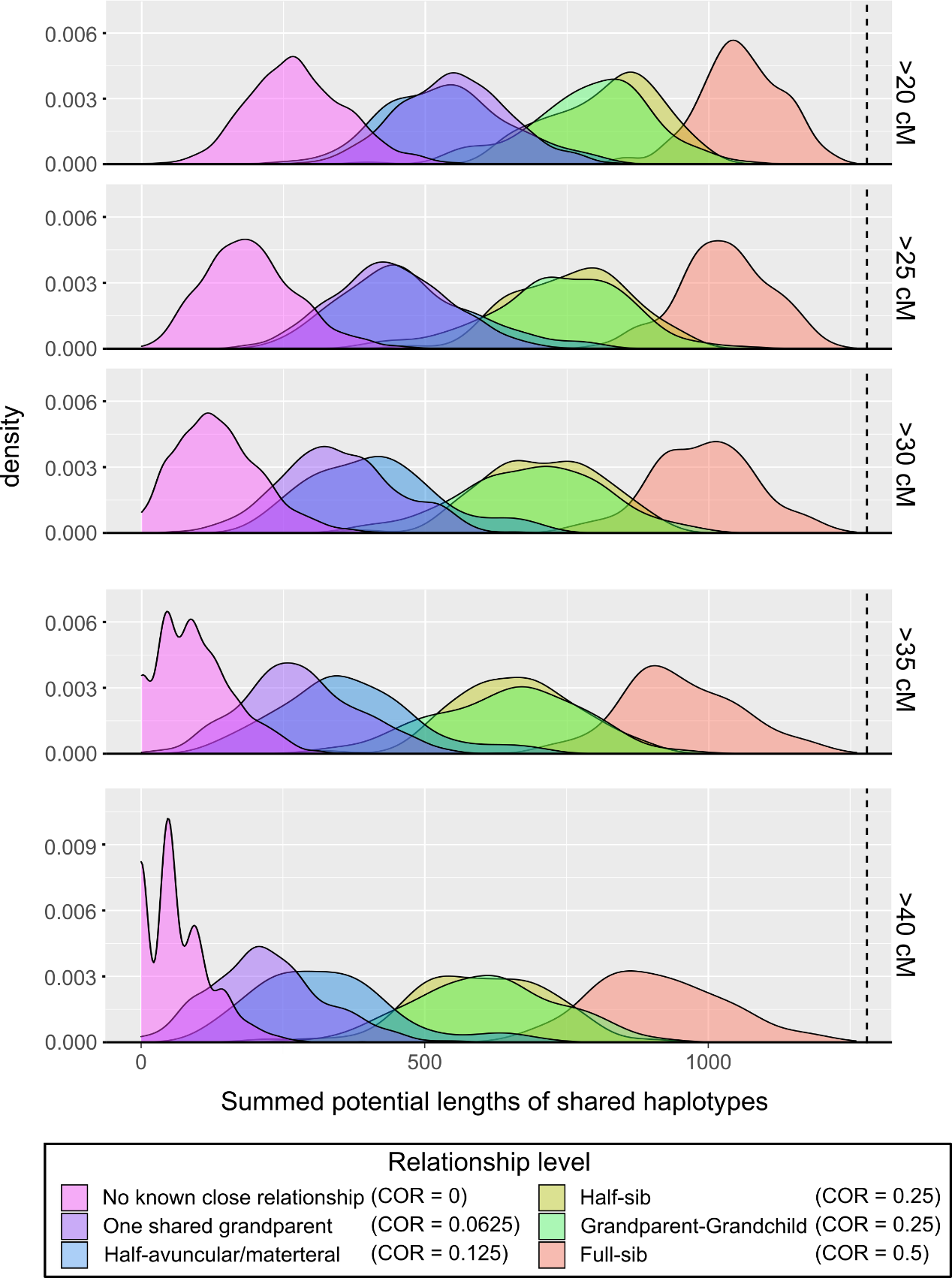

Supplement: Supplementary file 1 — Figure S1 [file 41438_2021_637_MOESM1_ESM.docx]
