# Supplementary material for: The use of shared haplotype length information for pedigree reconstruction in asexually propagated outbreeding crops, demonstrated for apple and sweet cherry
Source: Hortic Res. 2021 Sep 1;8:202. doi: 10.1038/s41438-021-00637-5 (PMC8408172; doi:10.1038/s41438-021-00637-5)

**Supplementary Figure S3.** Relationship probability estimates vs. summed potential lengths of shared haplotypes for pairs of cherry individuals.


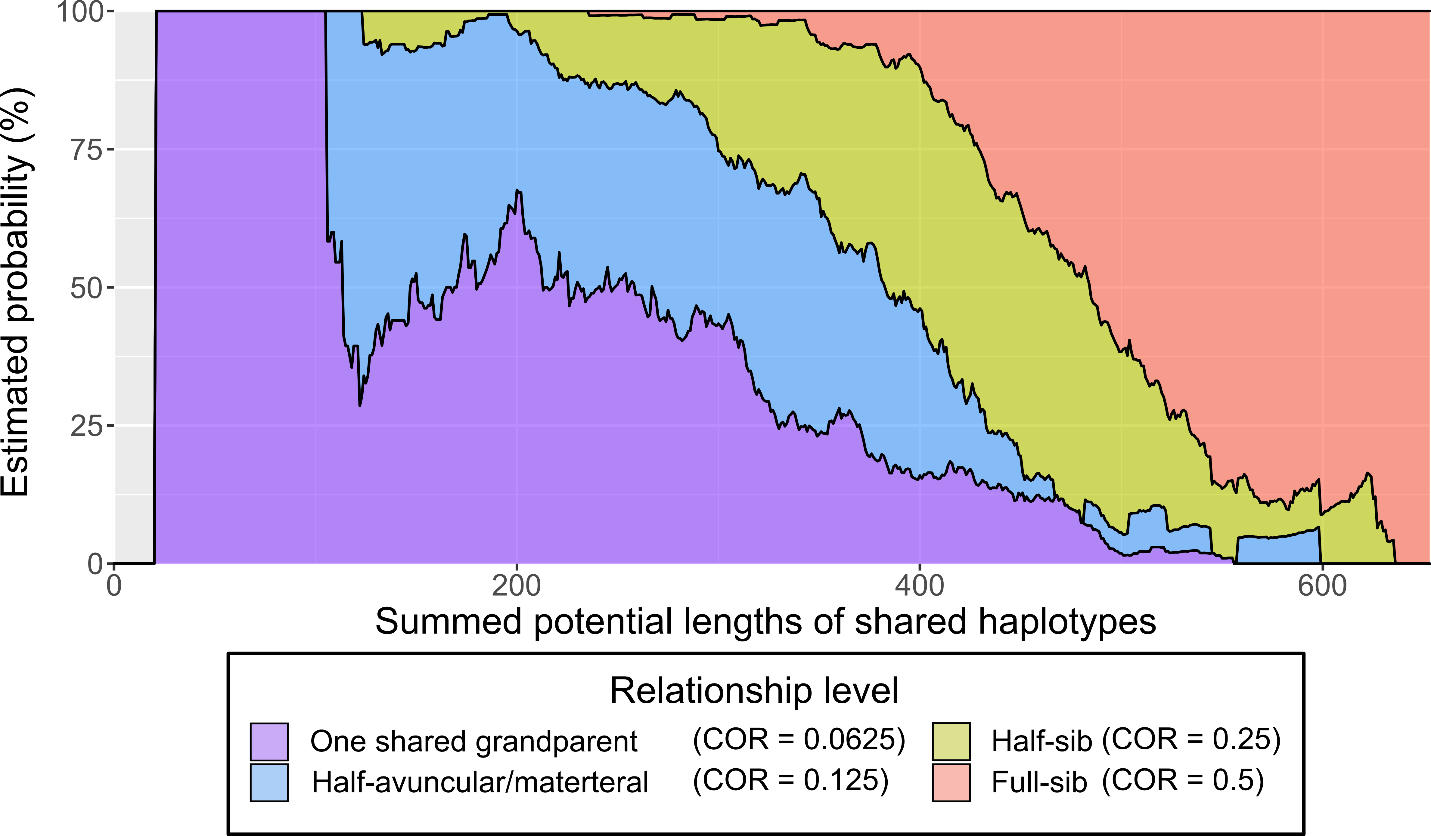

Supplement: Supplementary file 3 — Figure S3 [file 41438_2021_637_MOESM3_ESM.docx]
