# Supplementary material for: The use of shared haplotype length information for pedigree reconstruction in asexually propagated outbreeding crops, demonstrated for apple and sweet cherry
Source: Hortic Res. 2021 Sep 1;8:202. doi: 10.1038/s41438-021-00637-5 (PMC8408172; doi:10.1038/s41438-021-00637-5)
Supplement: Supplementary file 4 — File S1 [file 41438_2021_637_MOESM4_ESM.docx]

Discussion of case studies of pedigree reconstruction in apple and sweet cherry

*Case study 1. FSIB relationship (cherry: ‘Emperor Francis’ and ‘Schmidt’)*: Results were consistent with a FSIB relationship between ‘Emperor Francis’ and ‘Schmidt’, with evidence of additional recent shared ancestry between the two common parents indicated by excessive double IBS, much homozygosity, and coinciding homozygous regions (Figure 3). Historical records only mention partial ancestry for one of these cultivars, with ‘Schmidt’ being a seedling of ‘Festfleischige Schwarze Knorpelkirsche’ that arose about 1841 in Prussia^1^. The origins of ‘Emperor Francis’ were not known to early fruit historians but the cultivar was described by 1876 and apparently well known in France and England^1^. ‘Emperor Francis’ is a grandparent of the key self-fertile cultivar ‘Stella’, which puts it in the ancestry of most modern North American cultivars^2^ and was a parent of the F_1_ family used to establish the first genetic map of sweet cherry^3^ and to subsequently identify the largest-effect QTL for fruit size yet described for this crop^4,5^. ‘Schmidt’ was highly regarded a century ago especially for its large and brown rot-resistant fruit^1^ and is the parent of various lesser-known cultivars. A common set of attributes described by Hedrick^1^ highlight their currently demonstrated full-sib relationship: trees that are vigorous and productive and fruit that are large, firm, crisp, sweet, and highly/richly flavored.

*Case study 2. HSIB or GPGC relationship (cherry: ‘Van’ and ‘Windsor’)*: Results were consistent with a HSIB or GPGC relationship between ‘Van’ and ‘Windsor’, with evidence for additional recent shared ancestry. ‘Van’ was introduced in 1944 by the Agriculture and Agri-Food Canada breeding program in Summerland, British Columbia, Canada, recorded as being derived from open pollination of ‘Empress Eugenie’^7^. The small-fruited ‘Empress Eugenie’ is reported to be a “duke” cherry (*P. avium* × *P. cerasus*) that arose as a chance seedling in 1845 near Paris, France and was introduced to North America by 1877 and widely distributed^1^, although no tree has been found in North America or Europe (A. Iezzoni, pers. comm.) for DNA-based confirmation. The paternal parent of ‘Van’ was recently discovered^5^ as ‘Black Republican’, determined with SNP array data analyzed as described in Vanderzande et al.^8^. The parentage of ‘Windsor’ is unknown in the literature, and the cultivar was introduced in 1881 for commercial use as a pollinizer in Windsor, Ontario, Canada^1,7^. These cultivar histories are consistent with ‘Empress Eugenie’ being the shared parent of ‘Van’ and ‘Windsor’. Alternatively, ‘Windsor’ could be a grandparent of ‘Van’ via the latter’s missing parent if that missing parent is not actually ‘Empress Eugenie’. The patterns of haplotype sharing of phased data (results not shown) are indicative with a GPGC relationship, with six regions consistent with ‘Van’ inheriting recombined ‘Windsor’ homologs. Whatever its identity, the missing parent of ‘Van’ contributed to modern cultivars a positively selected large-fruit allele, which is homozygous in the most-produced late-season cherry of North America, ‘Sweetheart’^5^. The regions of homozygosity (Figure 4) might represent deficiencies in the array for detecting polymorphism or a high degree of endogamy in their shared genepool extending back many generations. Breeding bottlenecks from inbreeding coupled with selection for certain attributes might also have resulted in a high frequency of particular haplotypes in certain genomic regions^5,6^, exacerbating the degree of haplotype sharing in descendant cultivars. However, the homozygous regions of the cultivars of case studies 1 and 2 (Figures 4 and 5) were entirely different, and one of the regions in ‘Van’ was long (39 cM), indicating a greater likelihood that the observed patterns of homozygosity and excessive double IBS were outcomes of close pedigree relationships.. Recent pedigree relationships must unite the missing parent of ‘Van’ (‘Empress Eugenie’?), ‘Black Republican’, and ‘Windsor’, given the significant haplotype sharing observed between ‘Windsor’ and ‘Black Republican’, consistent with the report of Cai et al.^5^ that the latter pair of cultivars shared a rare extended haplotype at the fruit size QTL, and because the degree of haplotype sharing between ‘Van’ and ‘Windsor’ was sufficiently high and with enough double IBS to actually represent a full-sib relationship.

*Case study 3. Two grandparents via single missing parent of grandchild (cherry: ‘Stella’)*: Our results verified the recorded pedigree for ‘Stella’ being an offspring of the ungenotyped parent JI 2420, which was confirmed to be ‘Napoleon’ x ‘Emperor Francis’ (Figure 5). Although pedigree records existed for JI 2420^7^, pedigree records have sometimes been known to be incorrect, as demonstrated for the popular apple cultivar Honeycrisp apple^9^. In this case study, the recorded pedigree provided candidate grandparents that could fully account for the haplotypes of the missing parent. In future cases, SPLoSH results could be used to identify candidate grandparents without the presence of pedigree records. Muranty et al.^10^ reported the identification of grandparental pairs for the single unknown parents of 26 apple accessions by testing all possible combinations of cultivars in the dataset as possible grandparental pairs and using the observed Mendelian inconsistent errors rates as the test statistic for rejection or confirmation. While that method was successful, it was relatively computationally intense to test every combination, taking 18.5 days spread over multiple nodes of a supercomputer, and was restricted to only identification of grandparental pairs where one parent was already identified. The method in our study could be used to identify single grandparents (as well as other close relationships) without the need to have one parent available nor to have the other grandparent available, as demonstrated in case study 5. Two other apple studies reporting the identification of previously unknown grandparents were for the single grandparent of ‘Frostbite’^11^ and two grandparents of Co-op 7 identified separately^12^, using manual haplotype inspection and filtering of unphased SNP data, respectively. While such methods worked, identification of grandparents was not as streamlined as in the present study and required some provenance information that was not necessary in our approach.

*Case study 4. Large HSIB group sharing unknown parent (apple: 29 cultivars via “Unknown Founder 1”)*: The group of offspring of the now-imputed “Unknown Founder 1” included cultivars originating in Belgium, Germany, the United Kingdom, and some of ambiguous origin. The group contained several cultivars previously identified as ancestors of numerous other cultivars. ‘Ananas Renette’, ‘Baumann’s Renette’, and ‘Golden Noble’ were previously identified as parents of five, six, and nine cultivars, respectively, in Muranty et al.^10^. Our results also identified Unknown Founder 1 as a direct parent of ‘Sturmer Pippin’ and thereby as a grandparent of the major commercial cultivar Braeburn^10^.

The method used here for postulating and confirming the presence of a HSIB group and the deduction of the haplotype composition of its common unknown parent using SNP markers has to the best of our knowledge not been previously reported, at least in plants. A somewhat similar approach had been followed for the imputation of the SSR profile of the apple cultivar Schmidt’s Antonovka following a similar procedure has previously been reported^13^. However, no phasing and haplotyping was needed there thanks to the multi-allelic nature of SSR markers. Identification of likely HSIB groups using statistical analyses of genome-wide SNP data was also reported for *Eucalyptus nitens*^14^, although that study did not confirm the relationships.

Half-sib and grandparent-child relationships theoretically have the same expected SPLoSH and COR values. Hence, they cannot be distinguished based on individual pair-wise comparisons. In the case here, provenance information was not always conclusive due to being incomplete. For example, ‘Baumann’s Reinette’ is only known to be an old cultivar, being first recorded in 1811^15^. This information was not conclusive as some of the other possible half-sibs could be of similar age. In our approach, HSIB vs. GPGC could be distinguished by means of a genome-wide comparison between the individual in question and the imputed parent for the haplotype composition of their homologs. Offspring from the imputed parent would be expected to have homologs composed of recombinant haplotypes from the imputed parent. In contrast, the genetic contribution from a grandparent will never require a recombination between two homologs of the imputed parent and so if such a recombination would be required, the individual in question could not be a grandparent. Hence, in the case here, the imputation step was first used to demonstrate that SPLoSH information could identify HSIB groups and secondly to confirm that none of the potential half-sibs was instead a grandparent of the others through the unknown parent. The latter distinction between half-sibs and grandparents might not be problematic in open-pollinated breeding populations such as those of Klápště et al.^14^, where there is a better understanding of individual origin eras.

*Case study 5. Complex recent ancestry (apple: ‘Cox’s Pomona’)*:

In this case study, phased genotypic data was needed to identify candidate recent ancestors of the unknown parent of ‘Cox Pomona’ and for subsequent pedigree reconstruction (Figure 7), whereby homologs of the unknown parent were compared to the larger dataset. Phased data enabled precise establishment that a combination of the three most likely candidate ancestors could completely account for the homologs of the unknown parent. The phasing also enabled the generation ordering of these ancestors by providing more detailed information on the accumulated recombinations in the homologs ‘Cox’s Pomona’ inherited from its unknown parent. Such a pedigree reconstruction scenario, involving a previously unknown grandparent and two great-grandparents, has not been reported previously. Two of the three recent ancestors of ‘Cox’s Pomona’ identified in this study, ‘Reinette des Carmes’ and ‘Reinette Franche’, occurred frequently in other cultivar pedigrees too^10^. Thus, these new results connect the pedigree of ‘Cox’s Pomona’ to many other cultivars.

*Case study 6. Likely GPGC relationship (apple: ‘Fameuse’ and ‘McIntosh’)*: ‘Fameuse’ has often been proposed as a parent of ‘McIntosh’ or sometimes as an unknown relative, considering the close geographical and relative temporal origins of these cultivars and the distinct phenotypes of flesh whiteness and fruit shape and (e.g., ^16–18^). However, the exact relationship between the two cultivars had not been previously established. ‘Fameuse’ was recorded as having originated in the early 1700s or perhaps even earlier whereas ‘McIntosh’ originated in either 1796 or 1811^19^. Results were consistent with this hypothesis, as the analysis of phased genotypic data revealed multiple instances of recombinant haplotypes of ‘Fameuse’ in ‘McIntosh’, with recombinant haplotypes often containing only a single switch between ‘Fameuse’ homologs (Figure 7). This result excluded relationships such as HSIB and HAAM and instead, coupled with the SPLoSH value indicating a COR of 0.26, suggested that ‘Fameuse’ was a grandparent of ‘McIntosh’. However, the alternative hypothesis of another relationship, such as ‘Fameuse’ being a double great-grandparent of ‘McIntosh’, could not be ruled out. The greatest evidence of this alternative scenario was the high degree fragmented shared haplotypes sometimes present on the same homologs of ‘McIntosh’ and the large number of apparent recombinant ‘Fameuse’ haplotypes. Identification of a common shared ancestor in ‘Api’ accounted for some of this haplotype sharing and some of the short ‘Fameuse’ haplotypes in ‘McIntosh’. Hence, more evidence would be necessary to confidently rule out alternative pedigree hypotheses and confirm the GPGC deduction here.

*References*

1. Hedrick, U. P. *The cherries of New York*. (J.B. Lyon Company, state printers, 1915).

2. Choi, C. & Kappel, F. Inbreeding, Coancestry, and Founding Clones of Sweet Cherries from North America. *Journal of the American Society for Horticultural Science* **129**, 535–543 (2004).

3. Olmstead, J. W. *et al.* Construction of an intra-specific sweet cherry (*Prunus avium* L.) genetic linkage map and synteny analysis with the Prunus reference map. *Tree Genetics & Genomes* **4**, 897–910 (2008).

4. Zhang, G. *et al.* Fruit size QTL analysis of an F₁ population derived from a cross between a domesticated sweet cherry cultivar and a wild forest sweet cherry. (2009).

5. Cai, L., Voorrips, R. E., van de Weg, E., Peace, C. & Iezzoni, A. Genetic structure of a QTL hotspot on chromosome 2 in sweet cherry indicates positive selection for favorable haplotypes. *Mol Breeding* **37**, 85 (2017).

6. Pinosio, S. *et al.* A draft genome of sweet cherry (*Prunus avium* L.) reveals genome-wide and local effects of domestication. *The Plant Journal* **103**, 1420–1432 (2020).

7. Lapins, K. O. *Fruit tree cultivars in British Columbia*. (Canada, Dept. of Agriculture, 1977).

8. Vanderzande, S. *et al.* High-quality, genome-wide SNP genotypic data for pedigreed germplasm of the diploid outbreeding species apple, peach, and sweet cherry through a common workflow. *PLOS ONE* **14**, e0210928 (2019).

9. Cabe, P. R., Baumgarten, A., Onan, K., Luby, J. J. & Bedford, D. S. Using Microsatellite Analysis to Verify Breeding Records: A study of `Honeycrisp’ and Other Cold-hardy Apple Cultivars. *HortScience* **40**, 15–17 (2005).

10. Muranty, H. *et al.* Using whole-genome SNP data to reconstruct a large multi-generation pedigree in apple germplasm. *BMC Plant Biology* **20**, 2 (2020).

11. Howard, N. P. *et al.* Elucidation of the ‘Honeycrisp’ pedigree through haplotype analysis with a multi-family integrated SNP linkage map and a large apple ( *Malus* × *domestica* ) pedigree-connected SNP data set. *Horticulture Research* **4**, 1–7 (2017).

12. van de Weg, E. *et al.* Epistatic fire blight resistance QTL alleles in the apple cultivar ‘Enterprise’ and selection X-6398 discovered and characterized through pedigree-informed analysis. *Mol Breeding* **38**, 5 (2017).

13. Pikunova, A. *et al.* ‘Schmidt’s Antonovka’ is identical to ‘Common Antonovka’, an apple cultivar widely used in Russia in breeding for biotic and abiotic stresses. *Tree Genetics & Genomes* **10**, 261–271 (2014).

14. Klápště, J. *et al.* Exploration of genetic architecture through sib-ship reconstruction in advanced breeding population of Eucalyptus nitens. *PLOS ONE* **12**, e0185137 (2017).

15. Smith, M. W. G. National apple register of the United Kingdom. (1971).

16. Fisher, D. V. & Kitson, J. A. The Apple. in *Quality and Preservation of Fruits* 47 (CRC Press, 1991).

17. Way, R. D. *et al.* Apples (MALUS). in *Genetic Resources of Temperate Fruit and Nut Crops* 4 (1991).

18. Marini, R. P. *1995 Apple Variety Evaluations*. (1995).

19. Bussey, D. J. & Whealy, K. *The Illustrated History of Apples in the United States and Canada*. vols 1–7 (Jak Kaw Press, 2016).
