# Supplementary figures and images for: The use of shared haplotype length information for pedigree reconstruction in asexually propagated outbreeding crops, demonstrated for apple and sweet cherry
Source: Hortic Res. 2021 Sep 1;8:202. doi: 10.1038/s41438-021-00637-5 (PMC8408172; doi:10.1038/s41438-021-00637-5)

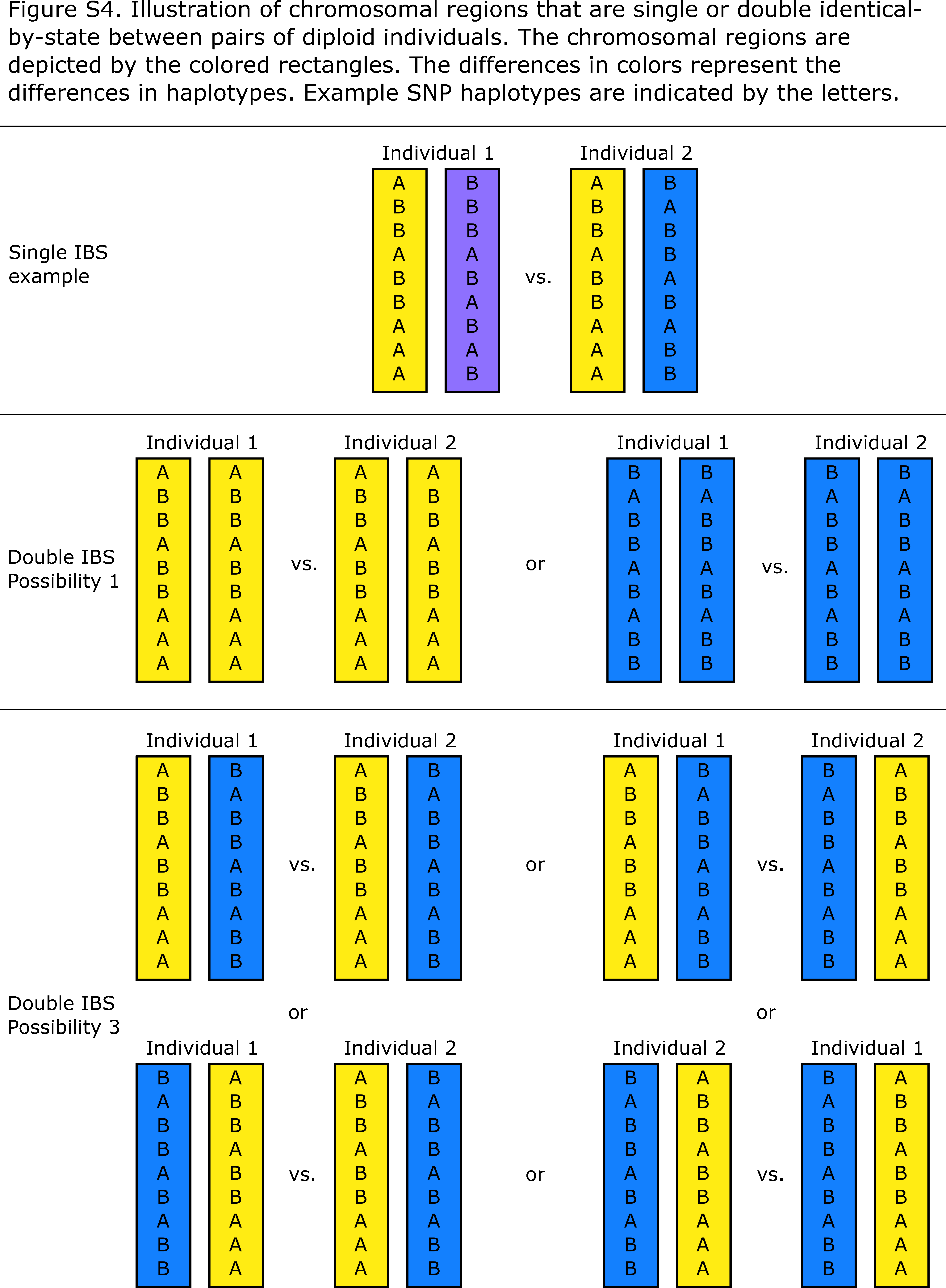

Supplement: Supplementary file 5 — Figure S4 [file 41438_2021_637_MOESM5_ESM.gif]
